# 2-Methyl-2,4-di-4-pyridyl-2,3-dihydro-1*H*-1,5-benzodiazepine acetic acid solvate

**DOI:** 10.1107/S1600536809049836

**Published:** 2009-11-25

**Authors:** Shi-Chao Wang, Qiu-Fei Hou, Shi-Mei Jiang

**Affiliations:** a State Key Laboratory of Supramolecular Structure and Materials, Jilin University, 2699 Qianjin Avenue, Changchun 130012, People’s Republic of China

## Abstract

In the title compound, C_20_H_18_N_4_·CH_3_COOH, the benzene ring forms dihedral angles of 81.34 (11) and 54.32 (11)° with the two pyridine rings. In the crystal, inter­molecular O—H⋯N hydrogen bonding links one 1,5-benzodiazepine mol­ecule and one acetic acid solvent mol­ecule into a dimer. These dimers, related by translation along the *b* axis, are further linked into chains *via* weak inter­molecular N—H⋯N hydrogen bonds.

## Related literature

For details of the synthesis and a related compound, see Hou *et al.* (2007 ▶).
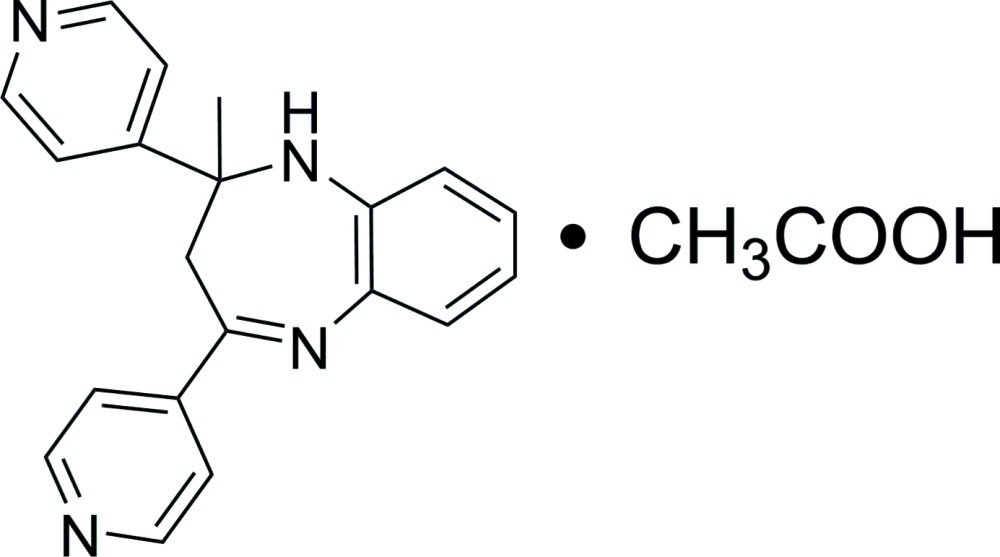


## Experimental

### 

#### Crystal data

C_20_H_18_N_4_·C_2_H_4_O_2_
*M* *_r_* = 374.44
Triclinic, 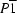

*a* = 8.925 (6) Å
*b* = 10.172 (8) Å
*c* = 12.283 (9) Å
α = 68.56 (3)°
β = 75.41 (3)°
γ = 88.52 (3)°
*V* = 1001.8 (12) Å^3^
*Z* = 2
Mo *K*α radiation
μ = 0.08 mm^−1^
*T* = 290 K
0.11 × 0.10 × 0.09 mm

#### Data collection

Rigaku R-AXIS RAPID diffractometer
Absorption correction: multi-scan (*ABSCOR*; Higashi, 1995 ▶) *T*_min_ = 0.991, *T*_max_ = 0.993
9946 measured reflections
4547 independent reflections
2400 reflections with *I* > 2σ(*I*)
*R*_int_ = 0.039

#### Refinement

*R*[*F* ^2^ > 2σ(*F* ^2^)] = 0.064
*wR*(*F* ^2^) = 0.211
*S* = 1.04
4547 reflections
256 parameters
H-atom parameters constrained
Δρ_max_ = 0.24 e Å^−3^
Δρ_min_ = −0.36 e Å^−3^

### 

Data collection: *RAPID-AUTO* (Rigaku, 1998 ▶); cell refinement: *RAPID-AUTO*; data reduction: *CrystalStructure* (Rigaku/MSC, 2002 ▶); program(s) used to solve structure: *SHELXS97* (Sheldrick, 2008 ▶); program(s) used to refine structure: *SHELXL97* (Sheldrick, 2008 ▶); molecular graphics: *PLATON* (Spek, 2009 ▶); software used to prepare material for publication: *SHELXL97*.

## Supplementary Material

Crystal structure: contains datablocks global, I. DOI: 10.1107/S1600536809049836/cv2662sup1.cif

Structure factors: contains datablocks I. DOI: 10.1107/S1600536809049836/cv2662Isup2.hkl

Additional supplementary materials:  crystallographic information; 3D view; checkCIF report

## Figures and Tables

**Table 1:** Hydrogen-bond geometry (Å, °)

*D*—H⋯*A*	*D*—H	H⋯*A*	*D*⋯*A*	*D*—H⋯*A*
N2—H2*A*⋯N4^i^	0.86	2.23	3.079 (4)	172
O2—H2*B*⋯N3^ii^	0.82	1.83	2.640 (4)	168

Symmetry codes: (i) 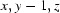; (ii) 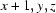.

**Fig. 1.**
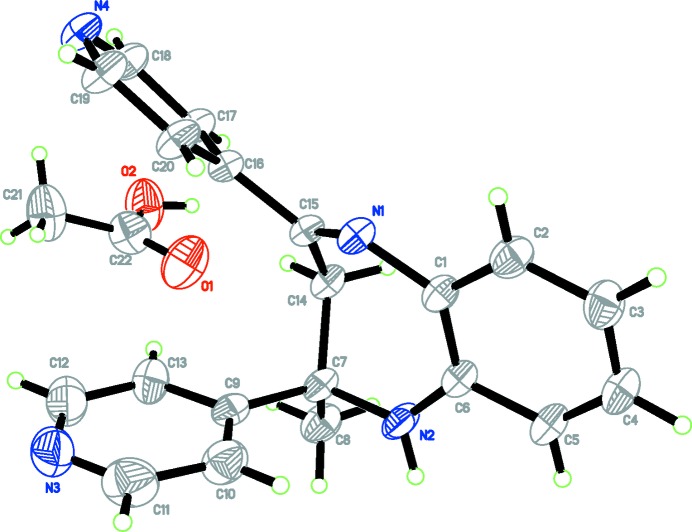
The asymmetric unit of the title compound showing the atomic numbering. Displacement ellipsoids of non-H atoms are drawn at the 30% probability level.

## supplementary crystallographic information

### Comment

The title compound (I) was unexpectedly obtained during our study of the Schiff base bis-pyridine complex (Hou *et al.*, 2007). In this paper, we report its crystal structure.

In (I) (Fig.1), the dihedral angles between benzene ring and two pyridine rings are 81.34 (11) ° and 54.32 (11) °, respectively. In the crystal structure, the intramolecular O—H···N and intermolecule N—H···N hydrogen bonds (Table 1) are observed.

### Experimental

The title compound and its single crystals suitable for the X-ray diffraction were prepared by slow evaporation of the ethanol solution which contains *o*-phenylenediamine, 4-acetylpyridine and a small amount of acetic acid at room temperatue.

### Refinement

All H atoms were placed in calculated positions (C—H 0.93 - 0.97 Å, N—H 0.86 Å, O—H 0.82 Å), and were included in the refinement in the riding model approximation, with *U*_iso_(H) = 1.2 or 1.5 *U*_eq_ of the parent atom.

### Crystal data

**Table:**

C_20_H_18_N_4_·C_2_H_4_O_2_	*Z* = 2
*M**_r_* = 374.44	*F*(000) = 396
Triclinic, *P*1	*D*_x_ = 1.241 Mg m^−^^3^
Hall symbol: -P 1	Mo *K*α radiation, λ = 0.71073 Å
*a* = 8.925 (6) Å	Cell parameters from 6051 reflections
*b* = 10.172 (8) Å	θ = 3.1–27.5°
*c* = 12.283 (9) Å	µ = 0.08 mm^−^^1^
α = 68.56 (3)°	*T* = 290 K
β = 75.41 (3)°	Block, colourless
γ = 88.52 (3)°	0.11 × 0.10 × 0.09 mm
*V* = 1001.8 (12) Å^3^	

### Data collection

**Table:**

Rigaku R-AXIS RAPID diffractometer	4547 independent reflections
Radiation source: fine-focus sealed tube	2400 reflections with *I* > 2σ(*I*)
graphite	*R*_int_ = 0.039
ω scans	θ_max_ = 27.5°, θ_min_ = 3.1°
Absorption correction: multi-scan (*ABSCOR*; Higashi, 1995)	*h* = −11→11
*T*_min_ = 0.991, *T*_max_ = 0.993	*k* = −13→13
9946 measured reflections	*l* = −15→15

### Refinement

**Table:**

Refinement on *F*^2^	Primary atom site location: structure-invariant direct methods
Least-squares matrix: full	Secondary atom site location: difference Fourier map
*R*[*F*^2^ > 2σ(*F*^2^)] = 0.064	Hydrogen site location: inferred from neighbouring sites
*wR*(*F*^2^) = 0.211	H-atom parameters constrained
*S* = 1.04	*w* = 1/[σ^2^(*F*_o_^2^) + (0.1067*P*)^2^ + 0.0338*P*] where *P* = (*F*_o_^2^ + 2*F*_c_^2^)/3
4547 reflections	(Δ/σ)_max_ < 0.001
256 parameters	Δρ_max_ = 0.24 e Å^−^^3^
0 restraints	Δρ_min_ = −0.36 e Å^−^^3^

### Special details

**Table:**

Experimental. (See detailed section in the paper)
Geometry. All e.s.d.'s (except the e.s.d. in the dihedral angle between two l.s. planes) are estimated using the full covariance matrix. The cell e.s.d.'s are taken into account individually in the estimation of e.s.d.'s in distances, angles and torsion angles; correlations between e.s.d.'s in cell parameters are only used when they are defined by crystal symmetry. An approximate (isotropic) treatment of cell e.s.d.'s is used for estimating e.s.d.'s involving l.s. planes.
Refinement. Refinement of *F*^2^ against ALL reflections. The weighted *R*-factor *wR* and goodness of fit *S* are based on *F*^2^, conventional *R*-factors *R* are based on *F*, with *F* set to zero for negative *F*^2^. The threshold expression of *F*^2^ > σ(*F*^2^) is used only for calculating *R*-factors(gt) *etc*. and is not relevant to the choice of reflections for refinement. *R*-factors based on *F*^2^ are statistically about twice as large as those based on *F*, and *R*- factors based on ALL data will be even larger.

### Fractional atomic coordinates and isotropic or equivalent isotropic displacement parameters (Å^2^)

**Table:**

	*x*	*y*	*z*	*U*_iso_*/*U*_eq_	
C1	0.2037 (3)	0.2500 (2)	1.0826 (2)	0.0436 (6)	
C2	0.1293 (3)	0.2017 (3)	1.2065 (3)	0.0533 (7)	
H2	0.0990	0.2679	1.2422	0.064*	
C3	0.0993 (3)	0.0601 (3)	1.2775 (3)	0.0603 (7)	
H3	0.0519	0.0310	1.3600	0.072*	
C4	0.1407 (3)	−0.0376 (3)	1.2242 (3)	0.0598 (8)	
H4	0.1206	−0.1338	1.2707	0.072*	
C5	0.2111 (3)	0.0060 (2)	1.1037 (3)	0.0545 (7)	
H5	0.2379	−0.0623	1.0698	0.065*	
C6	0.2455 (3)	0.1504 (2)	1.0273 (2)	0.0448 (6)	
C7	0.3831 (3)	0.3117 (2)	0.8120 (2)	0.0456 (6)	
C8	0.5137 (3)	0.2748 (3)	0.7236 (3)	0.0616 (8)	
H8A	0.5878	0.2247	0.7652	0.092*	
H8B	0.5639	0.3603	0.6590	0.092*	
H8C	0.4717	0.2163	0.6907	0.092*	
C9	0.2688 (3)	0.3983 (2)	0.7451 (2)	0.0476 (6)	
C10	0.1148 (4)	0.3542 (3)	0.7753 (3)	0.0701 (9)	
H10	0.0750	0.2681	0.8374	0.084*	
C11	0.0187 (4)	0.4432 (4)	0.7092 (4)	0.0911 (12)	
H11	−0.0858	0.4136	0.7294	0.109*	
C12	0.2161 (5)	0.6063 (4)	0.5942 (3)	0.0795 (10)	
H12	0.2526	0.6934	0.5325	0.095*	
C13	0.3180 (4)	0.5278 (3)	0.6521 (3)	0.0652 (8)	
H13	0.4216	0.5614	0.6290	0.078*	
C14	0.4524 (3)	0.4008 (2)	0.8686 (2)	0.0458 (6)	
H14A	0.5078	0.3404	0.9244	0.055*	
H14B	0.5263	0.4741	0.8050	0.055*	
C15	0.3307 (3)	0.4680 (2)	0.9343 (2)	0.0400 (5)	
C16	0.3302 (3)	0.6249 (2)	0.8930 (2)	0.0409 (5)	
C17	0.4648 (3)	0.7133 (2)	0.8320 (2)	0.0468 (6)	
H17	0.5584	0.6766	0.8080	0.056*	
C18	0.4577 (3)	0.8567 (2)	0.8072 (3)	0.0533 (7)	
H18	0.5499	0.9139	0.7690	0.064*	
C19	0.1991 (3)	0.8327 (3)	0.8883 (3)	0.0587 (7)	
H19	0.1058	0.8733	0.9053	0.070*	
C20	0.1949 (3)	0.6889 (3)	0.9202 (3)	0.0534 (7)	
H20	0.1013	0.6344	0.9601	0.064*	
C21	0.6804 (5)	0.8687 (5)	0.4824 (4)	0.1079 (15)	
H21A	0.5708	0.8432	0.5084	0.162*	
H21B	0.7133	0.9067	0.3955	0.162*	
H21C	0.7025	0.9387	0.5126	0.162*	
C22	0.7639 (4)	0.7424 (4)	0.5294 (3)	0.0768 (10)	
N1	0.2212 (2)	0.39891 (19)	1.02797 (19)	0.0445 (5)	
N2	0.3101 (3)	0.1793 (2)	0.9066 (2)	0.0551 (6)	
H2A	0.3069	0.1093	0.8836	0.066*	
N3	0.0682 (4)	0.5654 (3)	0.6210 (3)	0.0836 (9)	
N4	0.3286 (3)	0.9185 (2)	0.8342 (2)	0.0556 (6)	
O1	0.7070 (3)	0.6287 (3)	0.6004 (3)	0.1032 (9)	
O2	0.9124 (3)	0.7674 (3)	0.5022 (3)	0.1017 (9)	
H2B	0.9495	0.7015	0.5469	0.153*	

### Atomic displacement parameters (Å^2^)

**Table:**

	*U*^11^	*U*^22^	*U*^33^	*U*^12^	*U*^13^	*U*^23^
C1	0.0418 (12)	0.0344 (12)	0.0494 (14)	−0.0018 (10)	−0.0064 (11)	−0.0128 (10)
C2	0.0505 (14)	0.0459 (14)	0.0580 (17)	−0.0008 (11)	−0.0022 (13)	−0.0207 (12)
C3	0.0594 (16)	0.0541 (16)	0.0506 (16)	−0.0054 (13)	−0.0016 (13)	−0.0082 (13)
C4	0.0639 (17)	0.0381 (13)	0.0614 (18)	−0.0055 (12)	−0.0059 (15)	−0.0063 (12)
C5	0.0593 (16)	0.0318 (12)	0.0657 (18)	0.0025 (11)	−0.0113 (14)	−0.0136 (12)
C6	0.0443 (13)	0.0330 (11)	0.0535 (15)	0.0023 (10)	−0.0088 (12)	−0.0145 (10)
C7	0.0493 (13)	0.0320 (11)	0.0527 (14)	0.0030 (10)	−0.0068 (12)	−0.0167 (10)
C8	0.0634 (17)	0.0520 (15)	0.0663 (18)	0.0084 (13)	−0.0006 (15)	−0.0295 (14)
C9	0.0530 (15)	0.0407 (12)	0.0531 (15)	0.0034 (11)	−0.0099 (12)	−0.0247 (11)
C10	0.0612 (18)	0.0599 (17)	0.089 (2)	0.0034 (14)	−0.0206 (17)	−0.0266 (16)
C11	0.065 (2)	0.099 (3)	0.125 (3)	0.009 (2)	−0.034 (2)	−0.053 (3)
C12	0.089 (3)	0.074 (2)	0.073 (2)	0.0165 (19)	−0.028 (2)	−0.0203 (17)
C13	0.0704 (19)	0.0555 (16)	0.0571 (17)	0.0060 (14)	−0.0128 (15)	−0.0090 (13)
C14	0.0445 (13)	0.0332 (11)	0.0576 (15)	0.0035 (10)	−0.0088 (12)	−0.0175 (11)
C15	0.0426 (12)	0.0310 (11)	0.0477 (13)	0.0001 (9)	−0.0107 (11)	−0.0166 (10)
C16	0.0439 (12)	0.0337 (11)	0.0467 (13)	0.0007 (9)	−0.0102 (11)	−0.0178 (10)
C17	0.0439 (13)	0.0386 (12)	0.0554 (15)	−0.0006 (10)	−0.0055 (12)	−0.0193 (11)
C18	0.0523 (15)	0.0373 (13)	0.0640 (17)	−0.0072 (11)	−0.0067 (13)	−0.0167 (12)
C19	0.0472 (14)	0.0398 (13)	0.090 (2)	0.0043 (11)	−0.0119 (14)	−0.0291 (14)
C20	0.0448 (13)	0.0366 (12)	0.0772 (19)	−0.0015 (10)	−0.0091 (13)	−0.0235 (12)
C21	0.094 (3)	0.110 (3)	0.105 (3)	0.022 (2)	−0.039 (3)	−0.015 (3)
C22	0.0560 (18)	0.089 (2)	0.081 (2)	−0.0001 (17)	−0.0146 (17)	−0.028 (2)
N1	0.0446 (11)	0.0336 (10)	0.0524 (12)	0.0004 (8)	−0.0069 (10)	−0.0163 (9)
N2	0.0783 (15)	0.0291 (10)	0.0525 (13)	−0.0022 (10)	−0.0052 (12)	−0.0163 (9)
N3	0.104 (2)	0.0753 (19)	0.083 (2)	0.0293 (17)	−0.0411 (19)	−0.0328 (16)
N4	0.0589 (13)	0.0346 (10)	0.0743 (16)	0.0030 (10)	−0.0151 (12)	−0.0228 (10)
O1	0.0889 (18)	0.0776 (17)	0.108 (2)	−0.0144 (14)	0.0036 (16)	−0.0122 (16)
O2	0.0828 (17)	0.097 (2)	0.094 (2)	−0.0047 (14)	−0.0226 (15)	0.0014 (15)

### Geometric parameters (Å, °)

**Table:**

C1—C2	1.400 (4)	C12—C13	1.358 (4)
C1—C6	1.408 (4)	C12—H12	0.9300
C1—N1	1.409 (3)	C13—H13	0.9300
C2—C3	1.375 (4)	C14—C15	1.491 (3)
C2—H2	0.9300	C14—H14A	0.9700
C3—C4	1.373 (4)	C14—H14B	0.9700
C3—H3	0.9300	C15—N1	1.284 (3)
C4—C5	1.360 (4)	C15—C16	1.488 (3)
C4—H4	0.9300	C16—C20	1.381 (4)
C5—C6	1.415 (3)	C16—C17	1.387 (3)
C5—H5	0.9300	C17—C18	1.380 (3)
C6—N2	1.370 (4)	C17—H17	0.9300
C7—N2	1.452 (3)	C18—N4	1.325 (3)
C7—C8	1.522 (4)	C18—H18	0.9300
C7—C9	1.529 (3)	C19—N4	1.334 (3)
C7—C14	1.546 (4)	C19—C20	1.368 (4)
C8—H8A	0.9600	C19—H19	0.9300
C8—H8B	0.9600	C20—H20	0.9300
C8—H8C	0.9600	C21—C22	1.471 (5)
C9—C10	1.371 (4)	C21—H21A	0.9600
C9—C13	1.382 (4)	C21—H21B	0.9600
C10—C11	1.410 (5)	C21—H21C	0.9600
C10—H10	0.9300	C22—O1	1.194 (4)
C11—N3	1.309 (5)	C22—O2	1.290 (4)
C11—H11	0.9300	N2—H2A	0.8600
C12—N3	1.317 (5)	O2—H2B	0.8200
			
C2—C1—C6	119.0 (2)	C12—C13—H13	119.8
C2—C1—N1	112.7 (2)	C9—C13—H13	119.8
C6—C1—N1	128.2 (2)	C15—C14—C7	112.2 (2)
C3—C2—C1	122.5 (3)	C15—C14—H14A	109.2
C3—C2—H2	118.7	C7—C14—H14A	109.2
C1—C2—H2	118.7	C15—C14—H14B	109.2
C4—C3—C2	118.7 (3)	C7—C14—H14B	109.2
C4—C3—H3	120.6	H14A—C14—H14B	107.9
C2—C3—H3	120.6	N1—C15—C16	115.3 (2)
C5—C4—C3	120.2 (2)	N1—C15—C14	124.3 (2)
C5—C4—H4	119.9	C16—C15—C14	120.4 (2)
C3—C4—H4	119.9	C20—C16—C17	116.8 (2)
C4—C5—C6	123.1 (3)	C20—C16—C15	120.8 (2)
C4—C5—H5	118.4	C17—C16—C15	122.3 (2)
C6—C5—H5	118.5	C18—C17—C16	119.0 (2)
N2—C6—C1	126.6 (2)	C18—C17—H17	120.5
N2—C6—C5	116.9 (2)	C16—C17—H17	120.5
C1—C6—C5	116.5 (2)	N4—C18—C17	124.4 (2)
N2—C7—C8	107.2 (2)	N4—C18—H18	117.8
N2—C7—C9	112.2 (2)	C17—C18—H18	117.8
C8—C7—C9	109.8 (2)	N4—C19—C20	124.1 (2)
N2—C7—C14	109.4 (2)	N4—C19—H19	117.9
C8—C7—C14	109.2 (2)	C20—C19—H19	117.9
C9—C7—C14	108.97 (19)	C19—C20—C16	119.8 (2)
C7—C8—H8A	109.5	C19—C20—H20	120.1
C7—C8—H8B	109.5	C16—C20—H20	120.1
H8A—C8—H8B	109.5	C22—C21—H21A	109.5
C7—C8—H8C	109.5	C22—C21—H21B	109.5
H8A—C8—H8C	109.5	H21A—C21—H21B	109.5
H8B—C8—H8C	109.5	C22—C21—H21C	109.5
C10—C9—C13	117.3 (3)	H21A—C21—H21C	109.5
C10—C9—C7	122.5 (2)	H21B—C21—H21C	109.5
C13—C9—C7	120.2 (2)	O1—C22—O2	118.9 (3)
C9—C10—C11	117.9 (3)	O1—C22—C21	126.4 (4)
C9—C10—H10	121.0	O2—C22—C21	113.9 (3)
C11—C10—H10	121.0	C15—N1—C1	123.9 (2)
N3—C11—C10	123.7 (3)	C6—N2—C7	129.0 (2)
N3—C11—H11	118.1	C6—N2—H2A	115.5
C10—C11—H11	118.1	C7—N2—H2A	115.5
N3—C12—C13	123.3 (3)	C11—N3—C12	117.4 (3)
N3—C12—H12	118.3	C18—N4—C19	115.8 (2)
C13—C12—H12	118.3	C22—O2—H2B	109.5
C12—C13—C9	120.4 (3)		

### Hydrogen-bond geometry (Å, °)

**Table:**

*D*—H···*A*	*D*—H	H···*A*	*D*···*A*	*D*—H···*A*
N2—H2A···N4^i^	0.86	2.23	3.079 (4)	172
O2—H2B···N3^ii^	0.82	1.83	2.640 (4)	168

Symmetry codes: (i) *x*, *y*−1, *z*; (ii) *x*+1, *y*, *z*.
